# The Effects of Behavioral Modification Based on Client Center Program to Health Behaviors among Obese University Students

**DOI:** 10.5539/gjhs.v6n1p33

**Published:** 2013-10-08

**Authors:** Ungsinun Intarakamhang, Anan Malarat

**Affiliations:** 1 Behavioral Science Research Institute, Srinakharinwirot University, Bangkok, Thailand; 2 Faculty of Physical Education, Srinakharinwirot University, Bangkok, Thailand

**Keywords:** behavioral modification, self-efficacy, self-regulation, self-care, BMI, obese students

## Abstract

The objectives of this research were to examine the effectiveness of Health Behavioral Modification based on the Client Center Program (HBMCCP) and to study behavioral change in relation to self – efficacy, self- regulation, self-care behaviors and body weight. The sample was 59 undergraduate students, who were selected by cluster random sampling. 29 participated in the HBMCCP for 8 weeks, and were followed up 4 weeks after the program, and 30 students in the control group. Data was collected 3 times, before, immediately after and 4 weeks after the program, by 6 scale – questionnaires which had high reliability of Cronbach’s alpha-coefficient between .81 to .94. The stratified variables were psycho-social variables, being a positive attitude towards health behavior and social support. Data were analyzed by MANOVA and ANCOVA. Results showed that 1) Obese students in the experimental group with HBMCCP had self – efficacy, self- regulation and self-care behavior at immediately after and 4 weeks later program significantly higher scores than before the program (p<0.000). For body weights at immediately after program were significantly lower scores than before the program (p=0.02), 2) Obese students in the program had self – efficacy, self- regulation and self-care behavior scores at immediately after and 4 weeks after the program significantly higher than obese students in the control group (p=0.009) and significantly lower body weights than obese students in the control group (p=0.026), and 3) No three - way interaction among positive attitude towards health behavior, social support and the program was found, although there was a two-way interaction between positive attitude towards health behavior and the program (p=0.001) and effect size=0.272.

## 1. Introduction

Globally, one of the most serious public health challenges of the 21^st^ century is overweight and obesity in childhood increasing worldwide and growing incidence of type 2 diabetes (WHO, 2013). Rapidly changing dietary practices and a sedentary lifestyle have led to increasing prevalence of childhood obesity (5–19 years) in developing countries (Gupta et al., 2012). The risk factors caused by lifestyle changes, including, poor diet with excess caloric intake, a lack of fruits and vegetables, a lack of exercise, smoking, drinking and stress. The key factors that lead to chronic diseases such as diabetes, hypertension, cardiovascular disease, cancer, arthritis, and tendon, arteriosclerosis and thrombosis, respiratory disorders during sleep, disease of the gallbladder and severe mental disorders (Arthur, 2005). The National Health and Nutrition Examination Survey: NHANES of the United State of America found that the prevalence of adults with a BMI greater than or equal to 25 kg/m^2^ in the year 1999-2000 was 64.5 then increased to be 68.0 in the year 2007-2008 (Katherine et al., 2010). A study of the obesity among young Americans revealed that there were 32% are obese and 40% of those remain obese in adolescents and that 75 to 80% of obese adolescents will remain obese as adults. In 2004, health studies in the Asia Pacific region showed that the prevalence of overweight and obesity in Thailand was 50% higher compared to 14 countries and ranked fifth following Australia, Mongolia, Republic of Vanuatu, and Hong Kong (Division of Public Health Statistics, 2007). The obesity data in over 15 years old of Thai people in 2008 to 2009 found that nearly one-third of Thai men and almost half of Thai women were obese (BMI>25kg/m^2^) compared to the previous survey conducted in 2003 to 2004 which shows obesity was increasing markedly. The prevalence of obesity in women had increased from 34.4 to 40.7% and in men increased from 22.5 to 28.4%. For obesity prevalence in Thai newborn to 12 years old was 40% and in adolescent obesity was 30% in 2007. The survey also, found that Bangkok had the highest prevalence of obesity at 44.2% of population (Health Systems Research Institute, 2009)

The director of the Department of Health clearly indicates that in the past decade, Thai adolescent obesity, especially aged 15 years old or older is a growing problem. 58% of women had waist circumference over 80 cm whereas, 34% of males had a waistline over 90 cm. In addition, 30% of children, aged between 2-18 year old (17.6 million) are obese and this study could predict that 80% of these children will grow up into obese adults (Tul, 2010). Today, the health promotion is to reduce the virulence of chronic non-communicable diseases by focusing on the prevention and control of risk factors, including smoking, drinking alcohol, emotional health promoting behavior based on a good diet and nutrition less sugar, less salt, more fruits and vegetables and promoting physical activity to control for preventing obesity as well as creating a new attitudes (Nitaya & Nutcharee, 2009). Focusing on activities that use energy and get the body to the point of balance point is the way forward. Therefore, guidelines which seek to control, modify and promote new patterns of behavior by establishing the willingness and cooperation of the patient is necessary. This research applied technique that focused on client-centered theory (Rogers, 1987). There is a realization that to beat overweight problems the importance of HBMCCP program by client centered decision making that supports the personality and attitude of the client together with participatory learning through group processes is necessary. It helps achieve cooperation in the performance of the tasks and leads to the development of and changes in goals that are self-defined by the groups. The group members help to develop knowledge, skills, attitudes, and share their learning experiences helping each other in problem solving and builds confidence which carries over into daily life (Intarakamhang & Duangchan, 2012). This research studies the effects of the HBMCCP program for health behavioral modification that emphasizes the self-efficacy, self-regulation, self-care behaviors and body weight of the obese students. The results of this study will enable appropriate strategies to be developed to modify behavior to reduce the obesity and the other diseases.

## 2. Purpose of the research

To examine the effectiveness of HBMCCP in relation to self – efficacy, self- regulation, self-care behaviors and body weights in the experimental group by comparing those with the control group between before and after program and to studying the interaction between the psychosocial variables of, a positive attitude towards health behavior and social support.

## 3. Framework of experimental research

**Figure 1:**
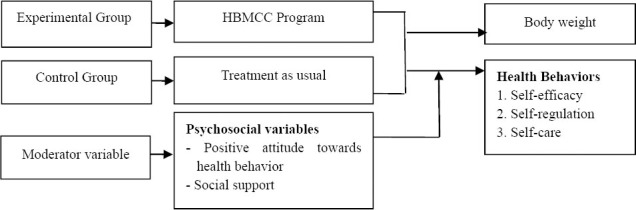
Framework of experimental research

The concept of the program developed from behavioral modification by the concepts of 1) Self-efficacy (Bandura, 1997) for establishing a successful model and the use of motivational words. 2) Self-regulation (Bandura, 1989), including the observation process, decision-making process and self-directed and 3) the concept of client-centeredness (Rogers, 1987) within a cooperative group. The research tested the efficacy of a health behavior modification program that emphasized client-centeredness in Figure 1.

## 4. Research Hypothesis

4.1 Obese students are trained with HBMCCP have scores of the health behaviors at a higher level and have body weight at lower level immediately after the trial and 4 weeks after the trial than before the trial.

4.2 Obese students in the experimental group have the health behaviors at a higher level and body weight at a lower level immediately after the trial and 4 weeks after the program than in the control group.

4.3 Students with high psychosocial characteristics, i.e. a positive attitude and social support when attending the HBMCCP will have high scores of self-efficacy, self-regulation and self-care behaviors too.

## 5. Research Methodology

### 5.1 Setting

The undergraduate students, who studying in the health science field, 220 students from the Faculty of Physical Education and Faculty of Nursing at Srinakharinwirot University, Thailand. The number of those overweight a BMI> 25 kg/m^2^ was 75 students or obesity prevalence was 34.09%, which is higher than the adolescent obesity prevalence in the population in Thailand.

### 5.2 Samples

#### 5.2.1 Sample Size

Undergraduate students whose body mass index ranged upwards from 25 kg/m^2^. Based on calculations by Nemet et al. (2005) 18 people per group is needed to detect reliable changes in body weight which are statistically significant at the 0.05 level. Thus, two groups of 30 students per each group were chosen in this study so as to discover any significant statistical differences associated with body weight.

#### 5.2.2 Randomization

The participants were sampling by cluster randomized controlled trial. Cluster Randomization and the students were divided into 2 groups who studying at Physical Education Faculty in the experiment group and studying at Nursing Faculty in a control group. This research was conducted in accordance with the standards of the Ethics Committee of Srinakharinwirot University. The certification number is SWUEC/EX50/2555 valid on November 5^th^, 2012. Participants meeting the following criteria were eligible to participate, 1) Undergraduate students having a body mass index ranging upwards from 25 kg/m^2^ (WHO, IASO; & IOTF, 2000), 2) Voluntary participation, with parental consent, 3) No medical conditions that would participate in the research, 4) Have not attended any behavioral modification for health programs in the past.

#### 5.2.3 Trial Period

Participants were available for a four month period from November 2012 to February 2013.

### 5.3 Research Design

The experimental research model with Factorial Design as follow in Table 1

**Table 1:** The experimental pattern with Factorial Design to study the relationship of the dependent variables

Sample group	Positive attitude towards health behavior	Social support
1.Experiment Group	High 15 people	High 15 people
	Low 14 people	Low 14 people
2.Control Group	High 15 people	High 15 people
	Low 15 people	Low 15 people
**Total**	59 people	59 people

### 5.4 The Experimental Pattern and Measurement

The research project took measurements before trial, immediately after trial at 8 weeks and 4 weeks after the completion of the trial at 12 weeks.

**Table:** 

Pretest	Treatment	Posttest 1	Posttest 2
Experimental group	O1, A, S, B, BMI X1	O2, B, Weight	O3 B, Weight
Control group	O1, A, S, B, BMI X0	O2, B, Weight	O3 B, Weight

O1 = Measuring before treatment, O2 = immediately after trial at 8 weeks

O3 = measurement 4 weeks after completed trial at 12 weeks

X1 = entered the HBMCC program, X0 = treatment as usual (not receiving the program)

A = Measurement of attitude towards health behavior, S = Measurement of social support,

B = Health assessment 3 Self behaviors, BMI = body mass index, Weight = Kilograms

### 5.5 Tools Used in this Research

#### 5.5.1 HBMCCP that Focuses on Client – Center

**Figure 2:**
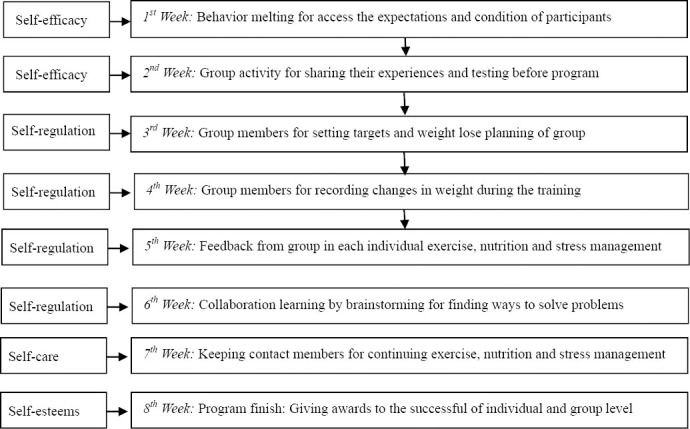
The process of HBMCCP that focuses on Client – Center

This program has been developed based on the theory of health behavior modification techniques and concepts learned through activities that focus on the service-centric processes and group processes together with the theory of self-efficacy, self-regulation and self –care. This program has been assessed for content validity by 3 highly experts in that area. The participants in the study were involved at all stage of the study, planning, setting goals and finally, taking responsibility for 8 activities by 2-3 hours/week as the process in details follow as Figure 2.

(a) The behavior melting; Assess the expectations and skills, the openness to accept the terms and conditions and extract stimuli that cause hunger, suppress the emotions that lead to overeating draw attention is to pick friend in their group to help them not to exceed their eating limits.
(b) Group activity, separated participants into 5 groups and the members of each group select 1 person to represent their group. At this point, the members were asked to introduce themselves to be familiar and share their experiences of being obese. Members help each other to calculate their body weight, height, and BMI and take a photo before commencing the program.
(c) Membership in a group was help to set targets and create a weight lose plan on their own initiative and crest menus that was adjusted to the correct caloric intake for each individual together with a modified plan and pattern of the exercise to burn calories to meet their goal.
(d) Group members helped to record changes in weight during the training and exercise including, eating and mood management. They arranged for the group meetings to monitor progress, weighting daily. A health behavior logbook was given to everyone and finally pathways to integrate the knowledge that has been gained from the sub-group to group as a whole were established.
(e) Member within the group were helped to familiarize themselves with topics such as, exercise, nutrition and stress management. Members of the group were trained on food selection skills, amount of food to lose weight that is suitable for themselves in accordance with their experience and verify its accuracy with feedback from the group.
(f) Collaboration skills and brainstorming. To find ways to solve problems. Review goals and plans for weight lose within the group and improve mental health and learning of all members of the group.
(g) Members were to encourage each other with the appreciative comments. Congratulate other members through, telephone calls, emails, Facebook that is coaching as a buddy. Keeping close contact to monitor and discuss problems within the group.
(h) Giving awards to the successful, at both an individual and group level to promote self-evaluation by group members.

#### 5.5.2 Treatment as Usual

Participants in the control group were given information regarding obesity upon the completion of the research.

#### 5.5.3 The Research Instruments Used to Collect Data

Tools used to collect data for this research were developed through a literature review of relevant research that focused on behavior modification for health programs based on self-efficacy theory and self-monitoring by Bandura (1989), and health assessment based on the measurement model of Intarakamhang (2009) and Supitcha et al. (2012) together with social support (House & Kahn, 1985) so as to create the content structure of the program. 3 experts tested for content validity. The instrument was tried out 50 students who were overweight but not participating in the study. The Cronbach's alpha coefficient of the questionnaire follows: 1) 28 items (Supitcha et al., 2012) for the self-care with the α equal to 0.81, 2) 31 items (Intarakamhang, 2009; Supitcha et al., 2012) for the self-regulation which α equal to 0.81, 3) 12 items (Intarakamhang, 2009; Supitcha et al., 2012) for self-efficacy with α equal to 0.89, 4) 18 items (Intarakamhang, 2012) for attitude towards health behavior with α equal to 0.93, and 5) 18 items (House & Kahn, 1985) social support with α equal to 0.94.

### 5.6 Data Collection

Being collected 3 times as before the trial, immediately after trial at 8 Weeks and 4 weeks after completed trial at 12 weeks.

### 5.7 Data Analysis

To test the hypothesis by determining the points before the trial as a covariate variable and test the interaction between the psychosocial variables and HBMCC Program predict health behaviors and body weight by using Repeated Measures ANCOVA analysis and Three-way MANOVA with Repeated Measures for the Multivariate F test of significance.

## 6. Results

### 6.1 The Study Supported the Hypothesis 1

The participants in the control group remained obese. It was found that a focus on self-efficacy, self-regulation, and self- care behaviors, those measured at 8 weeks and at 12 weeks was higher than before the trial. And body weight was lower Table 2 and 3.

**Table 2:** The analysis of One-way MANOVA with Repeated Measures of the variable of health behaviors separated by the trial period

Sources of Variance	Wilks’s Lambda (Λ)	Multivariate F test	df	p-value	Partial η^2^
Trial period	0.478	5.758	6	0.00*	0.239

* Statistically significant at p <0.05

**Table 3:** ANOVA analysis with Repeated Measures of self-efficacy, self-regulation and self-care behaviors and body weight of participants in the experimental group

Sources of Variance	Sum of Square	Df	Mean of Square	F-test	p-value	Partial η^2^
Trial period * Self-efficacy	71.655	2	35.828	1.180	0.315	.040
Trial period * Self-regulation	1779.885	2	889.943	2.220	0.118	.073
Trial period * Self-care	2517.057	2	1258.529	18.257	0.000*	.395
Trial period * Body weight	383.278	2	191.644	7.136	0.002*	.203

* Statistically significant at p <0.05

From Table 2 the trial period had significantly the main influence on the dependent variable with an effect size equal to 0.239. It therefore could be said that HBMCCP was affected health behaviors throughout the duration of the trial. This result supports the hypothesis 1 that the obese participants in the program group had self-efficacy, self-regulation and self-care behavior at 8 weeks and 12 weeks higher than before the trial in the study with statistical significance at 0.05 (Multivariate F test=0.478, F=5.758, p<0.00).

Table 3 shows that self-care behavior and body weight changed over the trial period, statistically significant at p <0.01 whereas, self-efficacy and self-regulation were not dependent on the trial period. In the program group, the body weight of the participants at 8 weeks and 12 weeks were lower, statistically significant at 0.05 (F=7.136, df=2, p=0.02), with the effect size equal to 0.203. Therefore, this result supports hypothesis 1.

Table 4 shows those students who are obese and received the program had the mean of health behavior between 4 weeks after completing the program and before trial, and was statistically significant (p <0.05)

**Table 4:** The average health behaviors as calculated in the program.

Trial period	Mean	Before trial	Immediately after trial	4 weeks after completed trial
Before trial	103.72	-	6.828*	13.172*
Immediately after trial	110.55		-	6.348*
4 weeks after completed trial	116.90			-

Table 5 shows that students who are obese and received the health behavior change program had mean body weight during the 4 weeks after the experiment (71.741 kg) with the before trial (75.886 kg) and immediately after the experiment (71.179 kg). Statistically significant differences at p <0.05.

**Table 5:** The average body weight for the undergraduate students over the period of the program

Trial period	Mean	Before trial	Immediately after trial	4 weeks after completed trial
Before trial	75.886	-	4.707*	4.145*
Immediately after trial	71.179		-	0.562
4 weeks after completed trial	71.741			-

### 6.2 The Study Also Supported the Hypothesis 2

The obese students in the program had self-efficacy, self-regulation, and self-care behaviors at 8 weeks and 12 weeks, higher than those in control group. And body weight was lower than the control group as shown Table 6-7

**Table 6:** Analysis of variance, multivariate two-way repeated measures (Two-way MANOVA with Repeated Measures) of the dependent variable considering both the program group and control group

Sources of Variance	Statistic Wilks’s Lambda (Λ)	Multivariate test	df	p	Partial η^2^
**Between Group**					
Program Group (A)	.812	4.250	3	.009*	.188
**Within the Group**					
Trial period (B)	.644	4.793	6	.001*	.356
Interaction (A*B)	.621	5.282	6	.000*	.379

**Table 7:** Analysis of Repeated Measures ANCOVA for body weight of participants in the program group over the trial period, controlled for the body weight of the students before the program

Source of Variance	Sum of Square	df	Mean of Square	F-test	p-value	Partial η^2^
Covariate (weight before trial)	12269.985	1	12269.985	451.309	0.000*	0.890
Experimental group - control group	142.190	1	142.190	5.230	0.026*	0.085
Error	1522.504	56	27.188			

Table 6 shows that obese participants in the program group had self-efficacy, self-regulation and self-care behavior at 8 weeks and 12 weeks higher than the control group with statistical significance at 0.05 (Multivariate F test =4.250, df=3, p=0.009) and effect size was equal to 0.188. This indicates that participants in the program group and the control group had an average difference between the dependent variables. Thus, this result supports hypothesis 2.

Table 7 shows that participants in the both groups after attending the program found that their mean body weight were statistically significantly different at (p<0.05) immediately after the completion of the trial. The average mean of body weight for participants in the program group was lower than the control group at 8weeks and 12 weeks. Additional, the participants in the program group had body weight at 8 and 12 weeks lower than those in control group with a statistical significance at 0.05 (F=142.190, df=1, p=0.026) and effect size is equal to 0.085.

### 6.3 Analysis Testing the Hypothesis 3

Found that participants having different psychosocial characteristics having attended the program at its completion had a different attitude regarding self-efficacy, self-regulation and self-care behavior. Follow as Table 8.

**Table 8:** The analysis of the three-way MANOVA of the average of the dependent variable to test the interaction of a good attitude towards healthy behavior and social support between the experimental groups – control group

Sources of variance	Wilks’s Lambda	Multivariate F test	p	Partial η^2^	
**Internal HBMCC Program**				
-Experiment group and Control Group	0.897	1.882	0.145	.103
-Positive attitude towards health behavior high-low	0.172	6.107	0.001*	.272
-Social support high-low	0.904	1.725	0.174	.096
**Two-way variances**					
-Program*Positive attitude towards health behavior	0.920	1.420	0.248	.080
-Program*Social support	0.963	0.630	0.599	.037
-Positive attitude on health behavior* Social support	0.889	2.034	0.121	.111
**Three-way variances**					
-Program* Positive attitude towards health behavior* Social support	0.970	0.500	0.684	.030

As shown in Table 8, considering only the variables it was found that the HBMCC program and the different levels of social support did not affect the result in the self-efficacy, self-regulation and self-care behavior. Whereas, a poor attitude towards the health behaviors and in the self-efficacy, self-regulation and self-care behavioral made a statistically significant difference at 0.05 (Multivariate F test=6.107, p=0.001) and the effect size is equal to 0.272. The variance of the two-way and three-way ANOVA was found that there was no interaction between the HBMCC program, social support and positive attitude towards health behavior. Thus, this result do not support hypothesis 3.

## 7. Discussion

The results of testing the 3 hypotheses indicate that the Health Behavior Modification program Client-centered are effective modifiers of health behaviors and the body weight of participants. Those entering the program had a weight decrease (p <.05) which is in agreement with the study of Arirat (2003) which found a health promotion program using a group process together with self-control helped the overweight to modify dietary behavior and physical activity resulting in a better body weight.

Also, when considering the activities in this research focused on learning by the program participants and positive reinforcement, friends helping friends using repeatedly words as *spell triggers and extracted* to achieve self-confidence. Their behavior is directed towards positive activities and good diet. Regular exercise and stress management. Which corresponds to the concept of Bandura (1997), who asserts that a person self-efficacy is the key element for people which determines how much they effort they need to work successfully (Bandura, 1989), using cognitive processes related self-efficacy and self- regulation to visualize success and better enable a person to act successfully (Annesi & Gorjala, 2010). The results of this study found a change in behavior at the conclusion of the trial and at the follow up point 4 weeks later, this was without the constant input of the program, except some stimulatory activity such as, providing reinforcement via social networks, and self-motivated friend helping friend, through positive reinforcement. These findings show that learning activities that focus on the patient are important as the participants are made aware of the importance of their self-care behaviors constantly and in a positive way. Participants were motivated by their peer group and witnessing their success together with their own enabled them to continue decreasing their body weight. Additionally, they influence each other to exercise and remind each other to eat right etc. This result is in accordance with the study of Intarakamhang (2012) which studied the effect of modifying health behavior in a group with a high risk of metabolic disease which studied the effect of the PROMISE program and evaluated it based on the CIPP Model, together with health behaviors, and indicators of health, such as, body mass index, blood pressure and waist circumference. There were 4,649 vulnerable aged from 15 years and over in Bangkok. It was found that after participating in the program participants had 3 Self behaviors higher than before with statistical significance and had health behaviors indicators such as, body mass index, blood pressure and waist circumference lower than prior to enrollment at a statistically significant level. The study by Boonroum (2005) studied the outcome of a health promotion program according to the theory of self-efficacy augmented by the learning focused on exercise for good health. The participants were 50 first year university students in the program group. The group had discussions, received an exercise for good health hand book, offering and demonstrating aerobics with video and finally, prompting from the researcher. After the trial was completed it was found that participants in the program showed that they were more aware of their self-efficacy regarding exercise and had positive expectations of exercise, and whilst doing an exercise the pulse rates were better than prior to attending the program at a statistically significant level.

In this study the self-regulation was found to changes over the program correlating with the duration of the trial. At this point, we could summarize that the program that based on the client-center, extract by prompting the group members. Weight loss goals which are based on the concept of Schunk & Zimmerman (1997) indicate that self-regulation is a process that encourages and supports the understanding of behavior and satisfaction in order to achieve set goals. The results were consistent with the study of Michael (2003) who studied self- regulation change in the prevention of obesity and eating disorders that can be treated with behavioral change, finding that interpersonal communication is an important technique in the control of body weight. This result was similar to the study of Jakicic (2003) who conducted a study regarding exercise and obesity. Jakicic found that exercise is the most important in strategy to control the body weight, rather than diet. Perhaps the exercise was an effective factor in weight control whilst the energy intake was restricted. This research had studied the effects of self-regulation program by the physicians those encouraging their obese patients to start exercising at least 150 minutes per week and make a call to follow up about the exercise and augmented with home visits to assess the actual progress. The results showed that participants can control their body weight over the long-term with exercise regime of 300 minutes per week.

The results of the hypothesis testing showed the interaction between good attitudes towards the healthy behavior program and outcome, based on the social cognitive theory of Bandura (1989). Bandura asserts that human behavior is not a result of environment forces and punishment only but human behavior is inextricably linked to an individual’s idea and sense of self. The results of Duangchan et al. (2010) found that a positive behavior supports a positive outcome for actions. A positive attitude is a strong intention to commit seriously to a behavior. On the contrary, if a person has a strong negative attitude the strong intention not to behavior in an efficacious manner will appear.

The social support variables were associated with behavior and that led to positive effects on health status. Persons who have adequate social support have reduced stress and are able to adapt well and have good health. In addition, social support can be predictor of quality of life. And the study of Kusuma (2007) that studied the effectiveness of weight loss programs by applying the concept of self- regulation together with strong social support for nurses. The study found social support for weight loss is associated with good practice to reduce weight and is statistically significant. In accordance with the previous research of Supitcha et al. (2012) that found that social support can predict health behaviors related to nutrition status of the Department of Health personnel with a statistical significance. A finding that accords with this study when considering only the main effect found that receiving social support affected to healthcare with statistically significant at 0.05 (F-test=4.979, p=0.03). However, it was no found to interaction significantly in the program based on the self-efficacy, self-regulation and behavioral modification for health. This means that the participants who received social support and those do not receive social support once joining the program both evidenced positive changes in health behaviors and body weight was not affected by the level of social support. So this program is not limited to those who received social support only. May be it be due to the participants those continued to lose weight as a result of internal factors that act as the driving force for participants.

## 8. Suggestions for Research

8.1 Health caring agencies should develop the ability to focus on health behavior that focuses on client-centered.

8.2 Before the activity / project the social context and reality of the risk group should be investigated, to come to an understanding of the way of life of patients as much as possible, so as to gain the trust and readily instigate the activity plan, monitoring and achieve results for both the service providers and service recipients.

8.3 Based on the research results it was found that a good attitude of the enrolled in the program is important and should be open to candidates who voluntarily participate in the program group and have good self-esteem to change health behavior and can act independently, joining in the program for better success.

8.4 Universities should have a policy to be Healthy Universities, giving extra curriculum time to the promotion of health for students, at least eight weeks to raise a students' health awareness.

8.5 Future research should study behavioral modification for health programs with different techniques and learning activities, such as individual activities that focus on teaching knowledge. Online activity should be studied in the multiple experimental groups.

## 9. Conclusion

The Health Behavioral Modification based on the Client Center Program for obese university students could be cause increasing of self – efficacy, self- regulation and self-care behaviors and the beneficial effect on body weight of reducing.
